# Safety and Glycemic Outcomes of the MiniMed 780G System with a Disposable All-in-One Sensor

**DOI:** 10.1177/15209156251368928

**Published:** 2025-08-18

**Authors:** Laura M. Nally, Jennifer L. Sherr, Satish K. Garg, Brynn E. Marks, Lori M. Laffel, Catherine Pihoker, Siham D. Accacha, James R. Thrasher, M. Jennifer Abuzzahab, John H. Reed, Laya Ekhlaspour, Sonali Belapurkar, Dorothy I. Shulman, Bhuvana Sunil, Sarah A. MacLeish, Kashif Latif, Gregory P. Forlenza, Kristin Castorino, Rayhan A. Lal, Bruce W. Bode, Frances E. Broyles, Anders L. Carlson, Benjamin U. Nwosu, John Shin, Haoxi Ma, Alysha Salbato, Toni L. Cordero, Yuri Treminio, Jennifer J. McVean, Andrew S. Rhinehart, Robert A. Vigersky, for the SUCCEED Study Group

**Affiliations:** 1 Yale University School of Medicine, New Haven, Connecticut, USA.; 2 Department of Pediatrics, Barbara Davis Center of Childhood Diabetes, Aurora, Colorado, USA.; 3 Children’s Hospital of Philadelphia, Philadelphia, Pennsylvania, USA.; 4 Joslin Diabetes Center, Boston, Massachusetts, USA.; 5 Department of Pediatrics, University of Washington, Seattle, Washington, USA.; 6 NYU Langone Hospital, Long Island, New York, USA.; 7 Medical Investigations, Inc., Little Rock, Arkansas, USA.; 8 Children’s Minnesota, Minneapolis, Minnesota, USA.; 9 Endocrine Research Solutions, Roswell, Georgia, USA.; 10 Department of Pediatrics, University of California, San Francisco, San Francisco, California, USA.; 11 College of Medicine, University of South Florida, Tampa, Florida, USA.; 12 Multicare Institute for Research and Innovation, Tacoma, Washington, USA.; 13 UH Case Medical Center, Cleveland, Ohio, USA.; 14 AM Diabetes and Endocrinology Center, Bartlett, Tennessee, USA.; 15 Sansum Diabetes Research Institute, Santa Barbara, California, USA.; 16 Stanford University School of Medicine, Stanford, California, USA.; 17 Atlanta Diabetes Associates, Atlanta, Georgia, USA.; 18 Rainier Clinical Research Center, Renton, Washington, USA.; 19 International Diabetes Center, Minneapolis, Minnesota, USA.; 20 Northwell Health, New Hyde Park, New York, USA.; 21 Medtronic, Northridge, California, USA.

**Keywords:** type 1 diabetes, A1c, time-in-range, advanced hybrid closed loop, automated insulin delivery, pediatric, adult

## Abstract

**Introduction:**

The present study assessed the impact of the disposable Simplera Sync™ sensor with the MiniMed™ 780G (MM780G) advanced hybrid closed-loop (AHCL) system on type 1 diabetes (T1D) glycemic metrics, insulin delivery, and safety.

**Materials and Methods:**

Youths (aged 7–17 years) and adults (aged 18–80 years) with T1D were enrolled in this single-arm, nonrandomized study at 24 sites in the United States. Participants began with an ∼2-week run-in period where hybrid closed-loop (HCL; auto basal only) or open-loop insulin delivery was used, followed by an ∼3-month study period with AHCL activated. Glycemic outcomes and insulin delivery during the last 6–7 weeks of the study, when settings were optimized at investigator’s discretion, were compared with the run-in. Glycemic outcomes with the use of recommended optimal settings (ROS, 100 mg/dL glucose target with a 2-h active insulin time) were explored.

**Results:**

Time in automation was high (>93%) and mean time in range (TIR) increased from 54.4% ± 15.7% to 71.4% ± 9.9% (*P* < 0.001) in youths and from 66.5% ± 12.6% to 80.2% ± 8.1% (*P* < 0.001) in adults, primarily due to reduced time above range. Youths had a slight increase in time below range (TBR <70 mg/dL) from 1.6% ± 1.7% to 1.9% ± 1.4% (*P* < 0.001), while adults had no significant difference in TBR. For ROS users, TIR was 74.7% ± 9.3% in youths and 83.8% ± 7.4% in adults. Throughout the study ∼60% of total daily insulin dose was automated (auto basal and auto correction) in both cohorts. There were two cases of severe hypoglycemia and one episode of diabetic ketoacidosis (not related to the device).

**Conclusions:**

MM780G use with the Simplera Sync sensor is safe and demonstrated improved glycemic outcomes in both pediatric and adult participants with T1D, compared with the run-in period.

## Introduction

Automated insulin delivery (AID) systems have dramatically changed the way people manage type 1 diabetes (T1D). AID systems leverage data from continuous glucose monitoring (CGM) to algorithmically modulate insulin delivery through an insulin pump with an algorithm continuously analyzing data and automatically adjusting insulin doses toward a system set point or target range. By directly addressing dynamically changing insulin needs, these devices more closely mimic physiological insulin delivery compared with manual insulin dosing and have led to remarkable improvements in glycemia^1–3^ and quality of life.^4^ The International Society of Pediatric and Adolescent Diabetes strongly recommends AID systems for youths with T1D.^5^ The American Diabetes Association (ADA) and the European Association for the Study of Diabetes, Diabetes Technology Working Group, have cited numerous benefits with this mode of insulin delivery.^6,7^

Several advancements in diabetes management technology have been made, including the development of extended infusion sets that are designed for up to 7 days of continuous wear, minimizing disruptions to daily life.^8^ Likewise, newer disposable CGM sensors with an embedded transmitter have been developed to improve ease of use. In July 2024, the Food and Drug Administration (FDA) approved the Medtronic stand-alone Simplera™ CGM system and, recently, the Simplera Sync sensor (April 2025) for use with the MiniMed™ 780G system (MM780G). The purpose of this study was to investigate the safety and performance of the MM780G advanced hybrid closed-loop (AHCL) system when used with the Simplera Sync sensor on glycemic outcomes in youths and adults with T1D.

## Materials and Methods

### Study design and participants

This multicenter, open-label, single-arm study enrolled youths (7–17 years of age) and adults (18–80 years of age) diagnosed with T1D across 24 investigational sites in the United States (Clinicaltrials.gov, NCT05714059). Institutional review board approval was obtained for each investigational center. Informed consent and assent, if applicable, were obtained in accordance with the Code of Federal Regulations (CFR) Title 21, Part 50; the California Experimental Subject’s Bill of Rights; and the Health Insurance Portability and Accountability Act, before initiation of the study. Medical oversight during the study was conducted by investigational center staff with appropriate medical training and a physician (principal investigator) or designee who has managed people with diabetes using both CGM and insulin pump therapy.

**FIG. 1.**
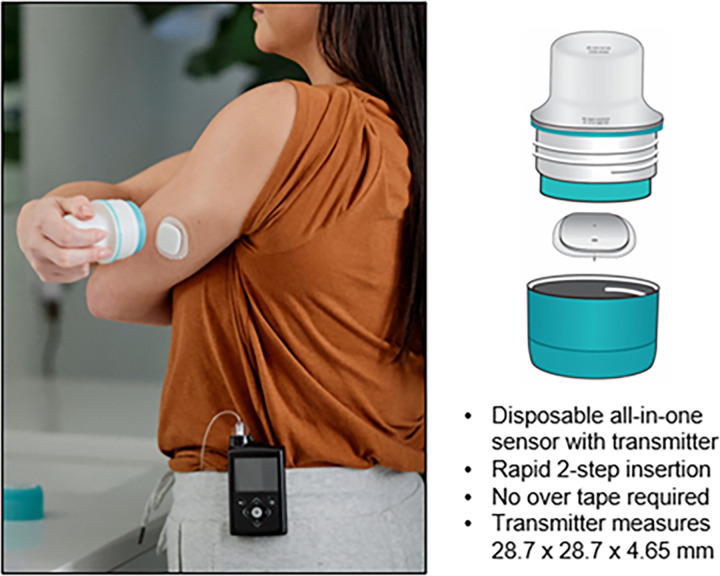
Simplera Sync™ sensor.

Participants used the MM780G system consisting of the MM780G pump (Medtronic, Northridge, CA, USA), the Extended™ 7-day infusion set (Medtronic), the disposable all-in-one Simplera Sync sensor (Medtronic), and the Accu-Chek® Guide Link blood glucose (BG) meter (Roche Diabetes Care, Inc., Indianapolis, Indiana, USA). The MM780G pump Bluetooth Low Energy 2.0 allowed communication between the Simplera Sync sensor and smartphone-compatible mobile app. The sensor has a rapid two-step insertion, measures half the size of the previous Guardian™ sensors, and requires no calibrations or overtape (Fig. 1). Youths were instructed to insert the sensor on either the back of the upper arm or upper buttock and adults were instructed to insert the sensor in the back of the upper arm only. The MM780G system provides automated delivery of basal and bolus insulin up to every 5 min to maintain glucose levels at or near a selected glucose target (GT) of 100, 110, or 120 mg/dL (with a temporary target of 150 mg/dL).

At Visit 1, participants provided consent or assent, were screened, and blood was collected for laboratories, before entering a run-in period of ∼2 weeks (Visits 2–6, Supplementary Data S1). At Visit 2, all participants received training and education on the study pump and sensor. During the run-in period, participants were able to use the system as a sensor-augmented pump. For individuals previously using a Medtronic pump with auto mode at enrollment, the use of auto basal (automated basal delivery only) was permitted, but auto correction (automated insulin correction) could not be started before the study period (Visit 7).

At Visit 7, participants began the study period that lasted ∼3 months (Visits 7–15) and were instructed to enable both auto basal and auto correction. The GT was set to 120 mg/dL and the recommended active insulin time (AIT) was 4 h, with a goal to titrate toward 2–3 h over ∼3 weeks. During the next 3 weeks, a GT of 100 mg/dL and an AIT of 2–3 h were recommended or could be set at the investigator’s discretion. During the final 6–7 weeks of the study period, the GT and AIT were adjusted according to what was deemed most suitable for each participant as determined by the investigator. Postintervention laboratory analysis for glycated hemoglobin (A1c) occurred at Visit 15 or at study exit.

### Inclusion/exclusion criteria

A comprehensive list of inclusion and exclusion criteria is shown in Supplementary Data S2. Briefly, general study inclusion criteria were a diagnosis of T1D for at least a year for participants aged 7–13 years and 2 years for participants aged 14–80 years. Additional criteria for inclusion were a minimum daily insulin requirement of at least 8 units per day; an A1c of <10% at screening; greater than 6 months of insulin pump therapy use with or without CGM; thyroid-stimulating hormone in the normal range (or if abnormal, a free T3 level below or within the reference range, and free T4 in the normal range); and a willingness to wear the MM780G system for the duration of the study.

Individuals were excluded from taking part in the study for any of the following criteria: a history of two or more severe hypoglycemic events that resulted in a coma, seizure, or hospitalization in the past 6 months; hospitalization or emergency room visit resulting in a primary diagnosis of uncontrolled diabetes in the past 6 months; diabetic ketoacidosis (DKA) in the past 6 months; or an inability to tolerate tape adhesive in the area of sensor placement or an unresolved adverse skin condition (e.g., psoriasis, dermatitis herpetiformis, rash, *Staphylococcus* infection) in the area of sensor placement. Participants with prior MM780G system experience before screening were excluded.

### Endpoints

The primary effectiveness endpoint was the mean percentage of time in range (TIR 70–180 mg/dL) during the last 6–7 weeks of the study period, compared with a predefined threshold derived from the pivotal trial^9,10^ of the MiniMed 670G system (TIR of 65.3%−7.5% for 7–17 years^9^ and TIR of 73.7%−7.5% for 18–80 years^10^) using a noninferiority test. The secondary effectiveness endpoints compared TIR with a predefined threshold (TIR of 65.3% for 7–17 years^9^ and TIR of 73.7% for 18–80 years^10^) using a simple superiority test and comparing the mean percentage of time spent in hypoglycemia <54 mg/dL with a predefined threshold (time below range [TBR] of 0.71% + 2.0% for 7–17 years^9^ and TBR of 0.86% + 2.0% for 18–80 years^10^), with the goal of demonstrating noninferiority.

Additional endpoints, for both groups, were analyzed by comparing the run-in period to the last 6–7 weeks of the study period and included mean sensor glucose (SG), glucose variability (standard deviation [SD] of mean SG and coefficient of variation of SG), percentage of TBR <54 mg/dL and <70 mg/dL, time in tight range (TITR, 70–140 mg/dL), and time above range (TAR, >180 mg/dL and >250 mg/dL) for the 24-h day, daytime (6:00 AM to 11:59 PM) and nighttime (12:00 AM to 5:59 AM). A comparison of insulin delivery metrics was also conducted. A subgroup analysis was performed for participants who used the recommended optimal settings (ROS), which consisted of a GT of 100 mg/dL and an AIT of 2 h for ≥95% of the time, during the last 6–7 weeks of the study period.

Per protocol, the primary safety endpoint was the overall mean change in A1c (%) from baseline to the end of study period. Additional safety outcomes included the incidence of severe hypoglycemia, DKA, serious adverse device effects, and unanticipated adverse device effects.

### Statistical analyses

For all endpoints, the intention-to-treat (ITT) population was used for evaluation and included all participants who started the study period. For normally distributed values, a one-sample *t-*test was conducted. For non-normally distributed values, a Wilcoxon signed-rank test was used. Differences between baseline/run-in versus end of study/6–7 weeks were tested with a significance level of 0.05 (two-sided). For noninferiority endpoints, the significance level was 0.025 (one-sided). Statistical analyses were performed using SAS™ 9.4 (SAS Institute, Cary, North Carolina, USA).

## Results

The study enrolled 250 individuals aged 7–80 years, with half of the participants (*N* = 125) in the pediatric cohort aged 7–17 years, and half (*N* = 125) in the cohort aged 18–80 years (Supplementary Data S3). In the pediatric group, there were seven screen failures, four early withdrawals before the start of the run-in period, and two withdrawals during the run-in period. During the study period, there were five withdrawals in the pediatric cohort; three were due to sensor issues, one participant had issues with the infusion set, and another chose to go back to the prestudy system. Thus, 107 pediatric participants completed the study.

In the adult group, there were 13 screen failures and 2 early withdrawals before the run-in period. Over the course of the study, there were five withdrawals; one withdrawal was due to technical issues with the pump, one participant was lost to follow-up, another had difficulty tolerating the infusion set, one experienced hyperglycemia and felt uncomfortable continuing, and another was withdrawn after receiving a steroid injection. A total of 105 adult participants completed the study. Baseline demographics and characteristics of the ITT population are provided in Table 1.

**Table 1.** Baseline Characteristics of the Intention-to-Treat Study Population

Characteristic	Pediatric (7–17 years) *N* = 112	Adult (18–80 years) *N* = 110
Age, years	13.3 ± 3.0	46.7 ± 15.8
Sex, *N* (%)		
Female	48 (42.9%)	56 (50.9%)
Male	64 (57.1%)	54 (49.1%)
Race, *N* (%)		
White	95 (84.8%)	104 (94.5%)
Asian, White	4 (3.6%)	0 (0.0%)
Asian, Native Hawaiian/Other Pacific Islander	0 (0.0%)	1 (0.9%)
American Indian or Alaska Native	0 (0.0%)	1 (0.9%)
American Indian or Alaska Native, Asian, White	1 (0.9%)	0 (0.0%)
American Indian or Alaska Native, White	1 (0.9%)	0 (0.0%)
Asian	2 (1.8%)	1 (0.9%)
Asian, Black or African American	1 (0.9%)	0 (0.0%)
Black or African American	6 (5.4%)	3 (2.7%)
Black or African American, White	1 (0.9%)	0 (0.0%)
Other (Moroccan)	1 (0.9%)	0 (0.0%)
Ethnicity, *N* (%)		
Hispanic or Latino	10 (8.9%)	5 (4.5%)
Not Hispanic or Latino	101 (90.2%)	105 (95.5%)
Not reported	1 (0.9%)	0 (0.0%)
Diabetes history, years	7.1 ± 3.8	26.0 ± 14.4
Weight, kg	57.7 ± 19.3	84.8 ± 19.5
BMI, kg/m^2^	21.9 ± 4.8	28.8 ± 5.9
BMI, z-score	0.6 ± 1.0	—
Treatment, *N* (%)		
Automated insulin delivery (pump + CGM + algorithm)	96 (85.7%)	82 (74.5%)
Sensor-augmented pump (pump + CGM)	10 (8.9%)	18 (16.4%)
Insulin pump (without CGM)	3 (2.7%)	10 (9.1%)
Multiple daily injections	2 (1.8%)	0 (0.0%)
Other	1 (0.9%)	0 (0.0%)
A1c, %	7.7 ± 1.0	7.4 ± 0.9
A1c, min–max	5.5–9.9	5.6–9.8

All data are shown as mean ± standard deviation (SD) or count (%).

BMI, body mass index; CGM, continuous glucose monitoring.

### Glycemic metrics, insulin delivered, and subgroup analyses

Glycemic metrics of the pediatric and adult groups, during run-in, the first 3 weeks at 120 mg/dL GT, the second 3 weeks at the 100 mg/dL GT, and the final 6–7 weeks with settings at investigator discretion are detailed in Tables 2 and 3, respectively. During the study period, AHCL was active >93% of the time on average for participants aged 7–17 years and >96% of the time on average for participants aged 18–80 years. From the first 3 weeks (GT at 120 mg/dL) to the second 3 weeks (GT at 100 mg/dL), there was an increasing trend in mean TIR and TITR for the overall 24-h period and a decreasing trend in the glucose management indicator (GMI) and TAR for both age groups. Similar trends in TIR and TITR and reduced mean SG and TAR were also observed for the daytime and nighttime periods (Tables 2 and 3). During the last 6–7 weeks of the study period, AHCL use was associated with increased mean TIR of 17% (baseline 54.4% to 71.4%) for participants aged 7–17 years (Table 2) and 13.7% (baseline 66.5% to 80.2%) for participants aged 18–80 years (Table 3).

**Table 2.** Pediatric Glycemic Outcomes Throughout the Study, Including Results of Recommended Optimal Settings Users

	Pediatric (7–17 years)						
	Run-in (*N* = 112)	120 mg/dL, AIT 2–4 h^a^ (*N* = 112)	100 mg/dL, AIT 2–4 h^a^ (*N* = 109)	Study^b^ (*N* = 109)	*P* run-in vs. study^b^	ROS^b,c^ (*N* = 41)	Non-ROS (*N* = 68)
Time in AHCL, %	—	96.2 ± 5.3	94.8 ± 8.4	93.5 ± 11.3	0.001^d^	96.9 ± 3.1	91.5 ± 13.8
A1c, %	7.7 ± 1.0	—	—	7.3 ± 0.8	<0.001^d^	7.0 ± 0.6	7.4 ± 0.9
GMI, %	7.6 ± 0.6	7.1 ± 0.4	7.0 ± 0.4	7.0 ± 0.4	<0.001	6.9 ± 0.4	7.1 ± 0.4
Mean of SG, mg/dL	180.4 ± 27.1	158.5 ± 15.7	153.1 ± 16.6	154.4 ± 17.6	<0.001	149.0 ± 15.3	157.6 ± 18.2
CV of SG, %	35.7 ± 4.8	35.1 ± 4.6	37.3 ± 5.1	37.3 ± 4.6	<0.001	36.8 ± 4.3	37.5 ± 4.8
%TBR <54 mg/dL	0.3 ± 0.6	0.3 ± 0.3	0.4 ± 0.4	0.4 ± 0.3	<0.001^d^	0.3 ± 0.4	0.4 ± 0.3
%TBR <70 mg/dL	1.6 ± 1.7	1.4 ± 1.1	1.9 ± 1.4	1.9 ± 1.4	<0.001^d^	1.9 ± 1.2	1.9 ± 1.4
%TITR 70–140 mg/dL	32.1 ± 14.1	45.6 ± 10.1	50.1 ± 9.6	49.2 ± 9.7	<0.001	52.7 ± 9.2	47.1 ± 9.4
%TIR 70–180 mg/dL	54.4 ± 15.7	70.3 ± 10.0	72.1 ± 9.5	71.4 ± 9.9	<0.001	74.7 ± 9.3	69.4 ± 9.8
%TAR >180 mg/dL	44.0 ± 16.1	28.2 ± 10.1	26.0 ± 9.6	26.7 ± 10.1	<0.001	23.3 ± 9.4	28.7 ± 10.1
%TAR >250 mg/dL	16.4 ± 11.1	8.1 ± 5.5	7.7 ± 6.3	8.0 ± 6.6	<0.001^d^	6.5 ± 5.3	9.0 ± 7.1
Daytime (6 AM–12 AM)							
Mean of SG, %	179.4 ± 27.5	160.9 ± 16.8	156.3 ± 18.0	157.4 ± 17.9	<0.001	152.4 ± 16.3	—
CV of SG, %	36.1 ± 5.1	35.9 ± 4.7	37.8 ± 5.1	37.4 ± 4.5	0.009	36.8 ± 3.9	—
%TBR <54 mg/dL	0.3 ± 0.7	0.3 ± 0.4	0.4 ± 0.5	0.3 ± 0.4	0.005^d^	0.3 ± 0.4	—
%TBR <70 mg/dL	1.7 ± 1.8	1.6 ± 1.3	2.1 ± 1.6	2.0 ± 1.4	0.002^d^	2.0 ± 1.4	—
%TITR 70–140 mg/dL	32.8 ± 14.2	43.9 ± 10.2	47.5 ± 9.8	46.2 ± 9.5	<0.001	49.0 ± 9.5	—
%TIR 70–180 mg/dL	54.8 ± 15.5	67.8 ± 10.7	69.3 ± 10.1	68.7 ± 10.0	<0.001	72.0 ± 9.7	—
%TAR >180 mg/dL	43.4 ± 16.2	30.6 ± 10.9	28.6 ± 10.4	29.2 ± 10.4	<0.001	26.0 ± 10.0	—
%TAR >250 mg/dL	16.2 ± 11.1	9.1 ± 6.1	8.6 ± 6.9	8.6 ± 6.8	<0.001^d^	7.0 ± 5.6	—
Nighttime (12 AM–6 AM)							
Mean of SG, %	183.3 ± 33.6	151.5 ± 17.1	143.6 ± 17.8	145.4 ± 21.2	<0.001	139.0 ± 17.0	—
CV of SG, %	32.3 ± 6.7	30.2 ± 6.8	33.0 ± 7.2	34.1 ± 6.8	0.020	34.3 ± 7.0	—
%TBR <54 mg/dL	0.3 ± 0.8	0.2 ± 0.4	0.3 ± 0.5	0.4 ± 0.5	0.006^d^	0.4 ± 0.4	—
%TBR <70 mg/dL	1.2 ± 2.3	0.9 ± 1.2	1.3 ± 1.6	1.6 ± 2.1	<0.001^d^	1.6 ± 1.6	—
%TITR 70–140 mg/dL	30.0 ± 16.9	50.5 ± 14.8	58.1 ± 15.2	58.0 ± 15.3	<0.001	63.6 ± 12.6	—
%TIR 70–180 mg/dL	53.1 ± 19.5	77.8 ± 11.9	80.7 ± 11.6	79.3 ± 12.6	<0.001	82.9 ± 10.3	—
%TAR >180 mg/dL	45.7 ± 19.7	21.2 ± 11.8	18.0 ± 11.0	19.1 ± 12.5	<0.001	15.5 ± 9.7	—
%TAR >250 mg/dL	17.0 ± 15.2	5.3 ± 5.5	5.0 ± 5.7	6.3 ± 7.2	<0.001^d^	5.0 ± 5.8	—

Data are shown as mean ± SD.

a Three weeks.

b The last 6–7 weeks of the study period. Glucose target and active insulin time were set at investigator’s discretion.

c Glucose target of 100 mg/dL and active insulin time of 2 h used independently for ≥95% of the time.

d Wilcoxon signed-rank test, otherwise *t* test.

AHCL, advanced hybrid closed loop; AIT, active insulin time; CV, coefficient of variation; GMI, glucose management indicator; ROS, recommended optimal settings; SG, sensor glucose; TAR, time above range; TBR, time below range; TIR, time in range; TITR, time in tight range.

**Table 3.** Adult Glycemic Outcomes Throughout the Study, Including Results for Those Who Used Recommended Optimal Settings

	Adult (18–80 years)						
	Run-in (*N* = 110)	120 mg/dL, AIT 2–4 h^a^(*N* = 109)	100 mg/dL, AIT 2–4 h^a^(*N* = 108)	Study^b^ (*N* = 107)	*P* run-in vs. study^b^	ROS^b,c^(*N* = 44)	Non-ROS(*N* = 63)
Time in AHCL, %	—	98.3 ± 2.8	97.8 ± 4.0	96.6 ± 6.6	<0.001^d^	97.5 ± 3.7	96.1 ± 8.0
A1c, %	7.4 ± 0.9	—	—	6.7 ± 0.6^e^	<0.001	6.6 ± 0.5^f^	6.9 ± 0.5^g^
GMI, %	7.2 ± 0.4	6.9 ± 0.3	6.7 ± 0.3	6.7 ± 0.3	<0.001	6.6 ± 0.3	6.8 ± 0.3
Mean of SG, mg/dL	161.0 ± 18.7	150.4 ± 12.6	141.2 ± 12.4	142.2 ± 12.8	<0.001	136.5 ± 12.0	146.2 ± 12.0
CV of SG, %	32.1 ± 5.0	29.8 ± 3.9	31.5 ± 4.4	31.9 ± 4.6	0.827^d^	31.1 ± 4.9	32.4 ± 4.3
%TBR <54 mg/dL	0.3 ± 0.5	0.2 ± 0.4	0.3 ± 0.3	0.2 ± 0.4	0.993^d^	0.2 ± 0.2	0.2 ± 0.5
%TBR <70 mg/dL	1.7 ± 1.9	1.1 ± 1.3	1.7 ± 1.5	1.5 ± 1.4	0.509^d^	1.7 ± 1.4	1.4 ± 1.4
%TITR 70–140 mg/dL	39.2 ± 13.0	48.5 ± 10.1	56.1 ± 10.1	56.1 ± 10.5	<0.001	61.6 ± 9.9	52.3 ± 9.1
%TIR 70–180 mg/dL	66.5 ± 12.6	76.7 ± 8.7	80.5 ± 8.0	80.2 ± 8.1	<0.001	83.8 ± 7.4	77.7 ± 7.7
%TAR >180 mg/dL	31.8 ± 13.1	22.2 ± 9.1	17.8 ± 8.3	18.2 ± 8.4	<0.001	14.5 ± 7.7	20.9 ± 7.8
%TAR >250 mg/dL	7.4 ± 6.1	3.9 ± 3.2	3.0 ± 2.9	3.4 ± 3.0	<0.001^d^	2.7 ± 2.6	3.9 ± 3.2
Daytime (6 AM–12 AM)							
Mean of SG, %	160.2 ± 18.9	151.7 ± 13.7	143.3 ± 13.2	144.6 ± 13.8	<0.001	137.9 ± 12.7	—
CV of SG, %	32.3 ± 4.9	30.4 ± 3.9	32.0 ± 4.5	32.2 ± 4.5	0.650^d^	31.8 ± 4.8	—
%TBR <54 mg/dL	0.3 ± 0.5	0.2 ± 0.4	0.3 ± 0.4	0.2 ± 0.4	0.537^d^	0.2 ± 0.2	—
%TBR <70 mg/dL	1.8 ± 2.0	1.2 ± 1.5	1.8 ± 1.7	1.6 ± 1.4	0.391^d^	1.8 ± 1.5	—
%TITR 70–140 mg/dL	39.7 ± 13.1	47.1 ± 10.7	53.8 ± 10.5	53.6 ± 11.3	<0.001	59.8 ± 10.4	—
%TIR 70–180 mg/dL	66.6 ± 12.6	75.1 ± 9.5	78.7 ± 8.4	78.2 ± 8.9	<0.001	82.3 ± 8.2	—
%TAR >180 mg/dL	31.6 ± 13.2	23.7 ± 9.9	19.5 ± 8.9	20.2 ± 9.2	<0.001	15.9 ± 8.4	—
%TAR >250 mg/dL	7.2 ± 6.0	4.3 ± 3.7	3.4 ± 3.2	3.8 ± 3.3	<0.001^d^	2.9 ± 2.8	—
Nighttime (12 AM–6 AM)							
Mean of SG, %	163.3 ± 24.0	146.2 ± 15.6	135.2 ± 15.7	135.0 ± 14.9	<0.001^d^	132.1 ± 15.2	—
CV of SG, %	29.7 ± 6.9	25.7 ± 5.5	27.4 ± 6.0	28.2 ± 5.9	0.030	26.7 ± 5.7	—
%TBR <54 mg/dL	0.3 ± 0.8	0.2 ± 0.6	0.2 ± 0.5	0.2 ± 0.6	0.086^d^	0.2 ± 0.4	—
%TBR <70 mg/dL	1.4 ± 2.6	0.9 ± 1.4	1.4 ± 1.9	1.4 ± 1.7	0.038^d^	1.4 ± 1.7	—
%TITR 70–140 mg/dL	38.0 ± 17.6	52.6 ± 16.3	62.7 ± 15.3	63.5 ± 13.9	<0.001	66.9 ± 14.6	—
%TIR 70–180 mg/dL	66.1 ± 17.2	81.5 ± 12.0	85.7 ± 10.7	86.2 ± 9.1	<0.001	88.3 ± 8.9	—
%TAR >180 mg/dL	32.5 ± 17.6	17.6 ± 12.0	12.9 ± 10.4	12.5 ± 9.1	<0.001	10.3 ± 9.1	—
%TAR >250 mg/dL	7.8 ± 8.8	2.6 ± 3.4	1.9 ± 3.4	2.2 ± 3.1	<0.001^d^	1.8 ± 2.8	—

Data are shown as mean ± SD.

a Three weeks.

b The last 6–7 weeks of the study period. Glucose target and active insulin time were set at investigator’s discretion.

c Glucose target of 100 mg/dL and active insulin time of 2 h used independently for ≥95% of the time.

d Wilcoxon signed-rank test, otherwise *t* test.

e *N* = 104.

f *N* = 42.

g *N* = 62.

AHCL, advanced hybrid closed loop; AIT, active insulin time; CV, coefficient of variation; GMI, glucose management indicator; ROS, recommended optimal settings; SG, sensor glucose; TAR, time above range; TBR, time below range; TIR, time in range; TITR, time in tight range.

Although not clinically significant, TBR <54 mg/dL and TBR <70 mg/dL increased slightly from 0.3% ± 0.6% to 0.4% ± 0.3% (*P* < 0.001) and from 1.6% ± 1.7% to 1.9% ± 1.4% (*P* < 0.001), respectively, in the pediatric group but was unchanged in adults. Both daytime and nighttime periods demonstrated improved glycemic metrics over time, with significant reductions in hyperglycemia and similar ROS impact on outcomes observed in both age groups (Tables 2 and 3).

Use of ROS was implemented by 41 of the pediatric participants (∼36%) and 44 of the adult participants (∼40%) (Tables 2 and 3). Pediatric ROS users achieved a TIR of 74.7%, while adults reached 83.8%, and their mean TAR >180 mg/dL was 23.3% and 14.5%, respectively. No appreciable difference in TBR was noted in either the pediatric or adult cohort using ROS, compared with the entire cohort during the last 6–7 weeks of the study. For ROS use over the daytime and nighttime periods, TIR and TITR increased even further and TBR <70 mg/dL was ≤2%. In contrast, when ROS were not used (non-ROS), the 24-h period TITR and TIR were lower, and GMI, mean SG, glucose variability, and TAR were higher (Tables 2 and 3). During this period, the GT of 100 mg/dL was used 93.5% of the time by the adult cohort and 79.8% of the time by the pediatric cohort. The AIT settings were more variable; however, the AIT setting of <3 h was used most of the time, with 50% and 61% use of the 2-h AIT by the adult and pediatric cohorts, respectively.

**FIG. 2.**
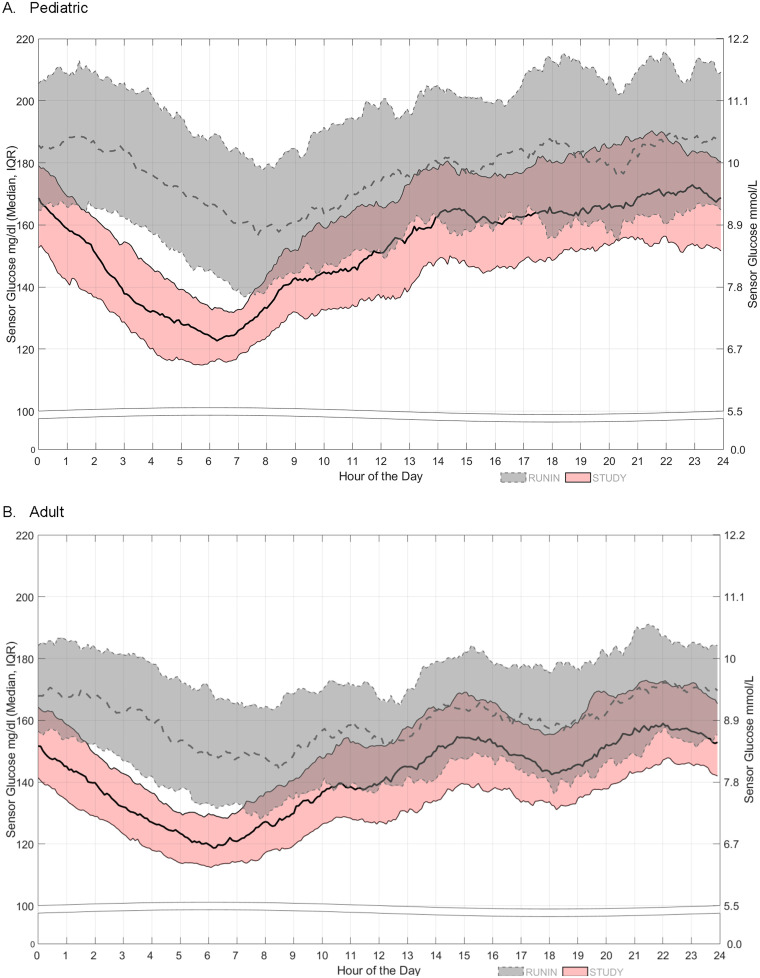
The 24-h day sensor glucose during the run-in and last 6–7 weeks of the study period. The median and interquartile range (IQR) of sensor glucose over a 24-h day during the run-in period (dashed line and gray band) and the last 6–7 weeks of the advanced hybrid closed loop (AHCL)-enabled study period (solid line and pink band) are shown for the **(A)** pediatric and **(B)** adult cohorts.

Figure 2 displays the median and interquartile range (IQR) of SG values across a 24-h day for both the pediatric and adult cohorts during the run-in and the last 6–7 weeks of the study. During AHCL use, the IQR of SG values was tighter compared with the run-in, with greater IQR tightening observed throughout the overnight and early morning hours. The mean ± SD of SG levels was significantly reduced from 180.4 ± 27.1 mg/dL during the run-in period to 154.4 ± 17.6 mg/dL with AHCL for pediatric participants. For adults, the mean ± SD of SG was 161.0 ± 18.7 mg/dL during run-in and was reduced to 142.2 ± 12.8 mg/dL with AHCL.

Insulin pump data, including daily grams of carbohydrate, carbohydrate entry, and insulin delivery during the run-in, and AHCL-use study phases for both age groups are shown (Tables 4 and 5). During the last 6–7 weeks of the study period, automated basal and automated correction insulin delivery was 41.0 ± 23.3 units/day (∼65% of total daily insulin dose [TDD]) for the pediatric cohort (Table 4) and 36.5 ± 22.0 units/day (∼61% of TDD) for the adult cohort (Table 5). Therefore, over 60% of the TDD for both groups was delivered automatically. In addition, the number of carbohydrate entries (i.e., meal boluses) decreased during the study period. The same insulin pump data and carbohydrate information are also shown for ROS and non-ROS users (Tables 4 and 5).

**Table 4.** Pediatric Daily Carbohydrate, Insulin Delivery, and Growth Characteristics

	Pediatric (7–17 years)						
	Run-in (*N* = 112)	120 mg/dL, AIT 2–4 h^a^ (*N* = 112)	100 mg/dL, AIT 2–4 h^a^ (*N* = 109)	Study^b^ (*N* = 109)	*P* run-in vs. study^b^	ROS^b,c^(*N* = 41)	Non-ROS(*N* = 68)
Daily CHO, g	184.7 ± 76.9	177.8 ± 65.8	170.8 ± 70.0	160.8 ± 68.8	<0.001^d^	164.8 ± 63.6	158.3 ± 72.1
Daily CHO entries, *N*	5.2 ± 2.1	5.0 ± 2.0	4.8 ± 2.0	4.5 ± 2.1	<0.001^d^	4.8 ± 2.0	4.3 ± 2.1
TDD, units	53.5 ± 26.0	59.6 ± 30.9	63.2 ± 32.7	62.5 ± 31.9	<0.001^d^	68.6 ± 35.1	58.9 ± 29.5
Total basal, units/day	21.4 ± 11.2	20.7 ± 11.5	23.7 ± 14.0	23.4 ± 13.0	0.0097^d^	24.5 ± 13.6	22.8 ± 12.7
Total bolus, units/day	32.1 ± 17.3	38.9 ± 20.3	39.5 ± 20.3	39.1 ± 20.0	<0.001^d^	44.1 ± 22.2	36.1 ± 18.1
Auto correction, units/day	—	15.2 ± 10.2	16.5 ± 10.7	17.6 ± 10.9	—	19.4 ± 11.8	16.5 ± 10.3
%Total bolus, %	—	38.5 ± 13.1	41.6 ± 14.2	44.4 ± 13.3	—	43.9 ± 13.5	44.7 ± 13.2
%TDD, %	—	24.7 ± 7.4	25.6 ± 7.5	27.4 ± 7.4	—	28.0 ± 7.6	27.1 ± 7.4
Auto basal + auto correction, %TDD	—	59.3 ± 10.9	62.6 ± 11.4	64.6 ± 10.8	—	63.3 ± 10.7	65.3 ± 10.9
Weight, kg	57.7 ± 19.3	—	—	59.8 ± 20.1^e^	<0.001^d^	61.0 ± 21.2	59.1 ± 19.6^f^
BMI, kg/m^2^	21.9 ± 4.8	—	—	22.6 ± 5.3^e^	<0.001^d^	22.8 ± 5.8	22.4 ± 5.1^f^
BMI z-score	0.6 ± 1.0	—	—	0.6 ± 1.0^e^	0.010^d^	0.6 ± 1.1	0.6 ± 1.0^f^

Data are shown as mean ± SD.

a Three weeks.

b The last 6–7 weeks of the study period. Glucose target and active insulin time were set at investigator’s discretion.

c Glucose target of 100 mg/dL and active insulin time of 2 h used independently for ≥95% of the time.

d Wilcoxon signed-rank test, otherwise *t* test.

e *N* = 108.

f *N* = 67.

CHO, carbohydrate; TDD, total daily insulin dose.

**Table 5.** Adult Daily Carbohydrate, Insulin Delivery, and Weight

	Adult (18–80 years)						
	Run-in(*N* = 110)	120 mg/dL,AIT 2–4 h^a^(*N* = 109)	100 mg/dL,AIT 2–4 h^a^(*N* = 108)	Study^b^ (*N* = 107)	*P* run-in vs.study^b^	ROS^b,c^(*N* = 44)	Non-ROS(*N* = 63)
Daily CHO, g	160.7 ± 76.3	163.9 ± 77.8	157.8 ± 78.9	152.2 ± 72.6	0.015^d^	165.4 ± 78.4	142.9 ± 67.3
Daily CHO entries, *N*	5.0 ± 2.2	5.2 ± 2.4	4.9 ± 2.2	4.8 ± 2.2	0.102^d^	5.0 ± 2.3	4.7 ± 2.1
TDD, units	54.7 ± 27.1	56.0 ± 29.1	57.9 ± 28.3	57.8 ± 28.0	<0.001^d^	57.7 ± 28.2	57.8 ± 28.1
Total basal, units/day	25.1 ± 13.8	22.7 ± 13.9	24.3 ± 14.1	23.8 ± 13.7	0.003^d^	23.0 ± 13.6	24.4 ± 13.9
Total bolus, units/day	29.6 ± 16.0	33.3 ± 17.2	33.7 ± 16.8	33.9 ± 16.1	<0.001^d^	34.7 ± 16.2	33.4 ± 16.2
Auto correction, units/day	—	10.7 ± 9.1	12.0 ± 8.9	12.7 ± 9.1	—	12.3 ± 9.3	13.0 ± 9.0
%Total bolus, %	—	30.9 ± 15.2	34.5 ± 15.8	36.0 ± 15.8	—	34.5 ± 16.7	37.0 ± 15.2
%TDD, %	—	17.7 ± 7.3	19.3 ± 7.5	20.7 ± 8.2	—	20.0 ± 8.3	21.1 ± 8.3
Auto basal + auto correction, % TDD	—	57.7 ± 13.1	60.7 ± 13.3	61.2 ± 13.0	—	59.2 ± 14.4	62.7 ± 11.9
Weight, kg	84.8 ± 19.5	—	—	85.1 ± 19.4^e^	0.070^d^	84.1 ± 18.4^f^	85.8 ± 20.2
BMI, kg/m^2^	28.8 ± 5.9	—	—	28.8 ± 5.8^e^	0.200^d^	28.3 ± 5.0^f^	29.1 ± 6.3

Data are shown as mean ± SD.

a Three weeks.

b The last 6–7 weeks of the study period. Glucose target and active insulin time were set at investigator’s discretion.

c Glucose target of 100 mg/dL and active insulin time of 2 h used independently for ≥95% of the time.

d Wilcoxon signed-rank test, otherwise *t* test.

e *N* = 106.

f *N* = 43.

BMI, body mass index; CHO, carbohydrate; TDD, total daily insulin dose.

**FIG. 3.**
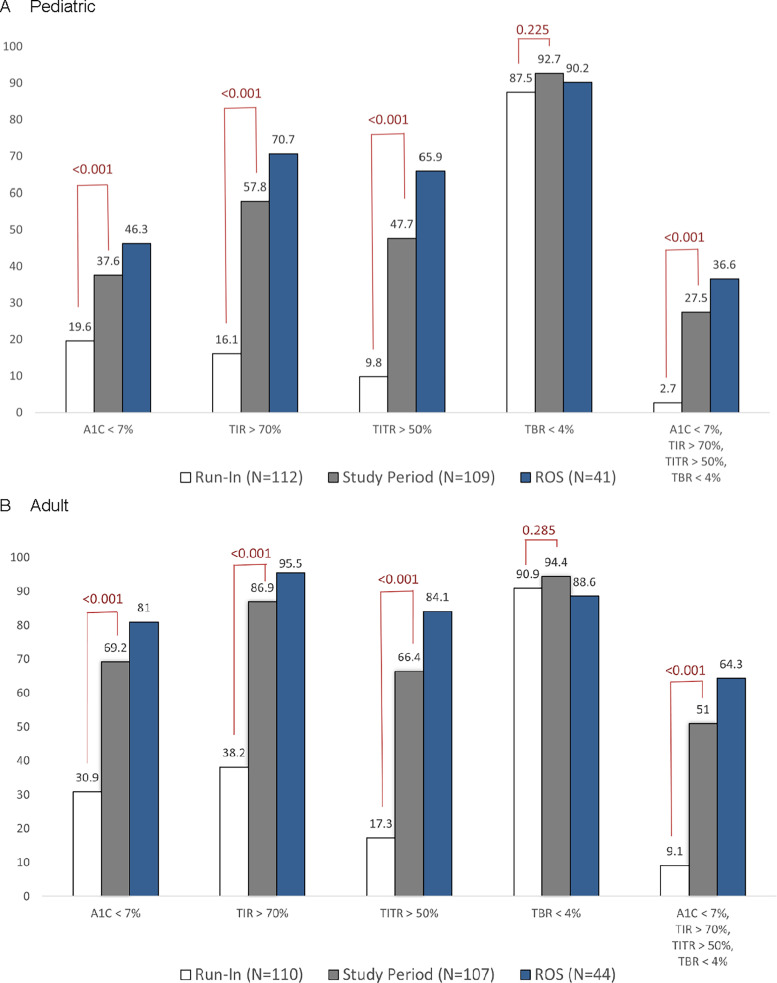
Percentage of participants meeting glycemic targets. The percentage of participants meeting A1c, time in range (TIR), time in tight range (TITR), and time below range (TBR) targets during run-in, the last 6–7 weeks of the study period, and use of recommended optimal settings (ROS); glucose target of 100 mg/dL with an active insulin time (AIT) of 2 h is shown for the **(A)** pediatric and **(B)** adult cohorts.

Participants achieving A1c <7%, TIR >70%, TITR >50%, and TBR <4% glycemic targets during the run-in and last 6–7 weeks of the study were compared (Fig. 3). In the pediatric group, the proportion of participants reaching an A1c <7% increased from 19.6% during the run-in to 37.6% at the end of the study. The adult group demonstrated similar glycemic improvements, where the proportion with A1c levels <7% more than doubled from 30.9% at baseline to 69.2% at study end. Of those using ROS, 46.3% of youth and 81% of the adult cohort achieved an A1c <7%.

### Safety

Compared with baseline, AHCL significantly reduced mean A1c from 7.7 ± 1.0 (*N* = 112) to 7.3 ± 0.8 (*N* = 109) for pediatric participants and from 7.4 ± 0.9 (*N* = 110) to 6.7 ± 0.6 (*N* = 104) for adult participants, meeting the primary safety endpoint. Throughout the study period, there were no serious adverse events (SAEs) reported for participants aged 7–17 years, and three SAEs for participants aged 18–80 years that included two severe hypoglycemia events and one DKA event, none of which was related to the investigational device. One participant experienced severe hypoglycemia, which included seizure activity after a user-initiated bolus and the participant declined transport to a hospital. Another participant experienced an episode of severe hypoglycemia and was found unresponsive after giving himself three manual injections, while disconnected from his pump and without checking BG. Regarding the episode of DKA, the participant did not replace an expired sensor for approximately 12 h, developed symptoms of DKA, and was driven to the emergency department by a family member. All participants recovered fully. In summary, there were no device-related episodes of severe hypoglycemia or DKA, and no serious or unanticipated adverse device effects.

## Discussion

In this prospective study, use of the Simplera Sync sensor as part of the MM780G system^9^ for 3 months was associated with improvements in glycemic metrics in youths and adults with T1D without a clinically significant increase in hypoglycemia. There were no SAEs in the pediatric cohort, but there were two episodes of severe hypoglycemia and one episode of DKA (unrelated to study device) in the adult cohort; rates of which are lower than those reported for adults with T1D.^11^ Overall, mean TIR using AHCL during the last 6–7 weeks increased with the pediatric cohort showing a 17% (∼4 h/day) increase, while the adults achieved 13.7% (∼3.3 h/day) more TIR from baseline. This change in TIR was accompanied by mean glucose that was substantially lower than during the run-in by 26 mg/dL in youths and ∼19 mg/dL in adults, a larger difference than that observed in the pivotal trial.^12,13^

Consistent time in closed loop is crucial to derive the full benefits that AID systems afford. The first-generation HCL from Medtronic, the MM670G, had many safety-related, system-mandated exits and frequent alarms, leading to discontinuation of device use by some.^14^ An increase in system automation was noted in the pivotal trial of the MM780G system compared with the MM670G system, with exits per week being only 1.1, 1.6, and 1.4 in the adult (*N* = 118), adolescent (*N* = 39), and youngest (*N* = 160) participants, respectively.^12,13^ In the present study, MM780G use with the Simplera Sync sensor resulted in 0.9 and 0.6 exits per week during the last 6–7 weeks of the study period for the pediatric and adult cohort, respectively. For both cohorts, approximately one-third of exits were due to “sensor expired,” “sensor updating,” or “AHCL disabled by user.” In addition, use of the sensor removes the need for a rechargeable transmitter, involves two-step insertion, and does not require overtape. Furthermore, paired use with the Extended infusion set that permits up to 7 days of wear (aligning with Simplera Sync sensor life) allows an individual to change devices once weekly, removing some of the onus of device burden.

While AID has long been demonstrated to improve glycemia in the overnight period, the present trial highlights that significant glycemic improvements were noted both during daytime hours (6:00 AM to 11:59 PM) and overnight during the last 6–7 weeks of the study period. In the pediatric cohort, TITR during daytime hours rose by 13.4% (from 32.8% ± 14.2% to 46.2% ± 9.5%, *P* < 0.001), while the adult cohort experienced a TITR increase of 13.9% (from 39.7% ± 13.1% to 53.6% ± 11.3%, *P* < 0.001). The greater time spent at normoglycemic levels was likely driven by automated boluses, which accounted for more than 27% and 20% of the TDD in youths and adults, respectively. By detecting missed meal boluses, the MM780G algorithm can mitigate postprandial hyperglycemia with more aggressive autocorrection boluses up to every 5 min,^15,16^ and potentially reduce the burden of more frequent user-initiated insulin doses. With the MM780G and Simplera Sync sensor, overnight TIR increased by 26.2% for youths and 20.1% for adults, whereas in the pivotal trial, overnight TIR increased by 18.1% in youths^12^ and 9.1% in adults.^13^

In the one-third of the pediatric cohort that used ROS, TIR approached ∼75%, while in adults (∼40% of the cohort), the TIR achieved was ∼84%. ROS were also associated with TITR >50% for both pediatric and adult participants. Similar results have been published in real-world data from youths and adults using the MM780G in Europe, the Middle East, and Africa.^17^ In the present study, these glycemic benefits were not observed in an exploratory analysis of non-ROS users. While there is no formal recommendation by the ADA for TITR, data from individuals without diabetes suggest that physiological glucose levels rarely rise above 140 mg/dL,^18^ and that this normoglycemic metric may be increasingly used as a goal.

When considering adoption of ROS, differences were noted in system settings between pediatric and adult participants. While the majority of study participants used the GT of 100 mg/dL, fewer youths adopted the lowest system set point. Conversely, the frequency of setting an AIT of 2 h was higher in the pediatric cohort. Personalization of device settings is critical and both of these tunable parameters provide opportunity for individualization of insulin delivery; yet, recognizing the age-based differences in system settings, providers may wish to consider approaching ROS through joint decision-making with people with diabetes for whom they care.

Interestingly, compared with run-in, the pediatric cohort entered fewer total grams of carbohydrates per day and a lower number of daily meal boluses, during study AHCL use; yet, they had an increase in TDD. This likely reflects meals or snacks that went unannounced, which the algorithm could account for by augmenting insulin delivery. Indeed, more than 40% of the daily bolus dose comprised automated corrections for youths. In contrast, adults had more consistent carbohydrate entry during the run-in and study periods, ∼160 versus ∼152 g, respectively. Despite this, 36% of the adult total bolus dose was due to automated insulin corrections. Therefore, regardless of meal-bolusing engagement, AHCL drove improved glycemia due to the additive benefit of frequent algorithm-automated basal and correction doses.

Over the course of the study, the pediatric cohort gained weight, which is expected as growth and development are hallmarks of childhood, especially for pubertal participants who are expected to experience increases in muscle mass and bone density. As such, the body mass index (BMI) z-score remained unchanged. Weight changes may be secondary to improvements in glycemia. Data from the first year after randomization in the Diabetes Control and Complications trial demonstrated that those with the greatest decrement in A1c levels had the highest increases in weight.^19^ When categorized based on whether weight gain was >2.5 kg, a clear picture emerges. For those, in the present study, who had <2.5 kg of weight gain, the mean change in A1c was −0.3%. For the 33 youths who exceeded the threshold of ≥2.5 kg weight gain, the mean change in A1c was −0.8%. Fewer than half (45%) of these participants with more weight gain had a BMI that categorized them as overweight. While some degree of weight gain is expected as A1c levels decrease, openly discussing the potential for weight gain in youth with suboptimal glycemia may help mitigate youth perceptions of body dysmorphia.

While the study was designed to engage youths and adults from diverse backgrounds, individuals identifying as White comprised nearly 85%−95% of the cohort, limiting generalizability to ethnic and racial minorities. Another limitation is that individuals with A1c >10% were excluded from enrollment, although there is evidence that AID can substantially benefit individuals with T1D and elevated baseline A1c.^20^ Notably, 17.8% of the pediatric cohort and 41.8% of the cohort were using HCL at baseline, which may reflect higher adoption rates than that in a general population living with T1D. Yet, inclusion of these individuals may have limited our ability to see more robust changes in glycemic metrics. Another limitation of the study was the variability in weight reporting, which was done either in-clinic or by participant self-report. Lastly, the nonrandomized study design (including ROS vs. non-ROS use randomization) may have presented confounding or selection bias. Strengths of this study are the inclusion and analysis of over 100 pediatric and adult participants, across multiple investigational centers. In addition, the exploratory analysis of ROS and the use of a new sensor contributed to the improved outcomes observed in this study. While more than half of the participants did not implement ROS, its use allowed the AHCL algorithm to drive even greater glycemic improvements.

In this trial, the Simplera Sync sensor used in conjunction with the MM780G system performed well, with participants achieving both lower A1c levels and higher TIR and TITR than previously reported in the MM780G pivotal trial, similar to large real-world data analyses.^17^ With AID systems improving glycemic metrics while reducing diabetes burden, it is time to ensure that all individuals with T1D are informed of this technology and have improved access to and coverage of these devices.^21^

## SUCCEED Study Group

AM Diabetes and Endocrinology Center (Bartlett, Tennessee, USA): Kashif Latif, MD. Atlanta Diabetes Associates (Atlanta, Georgia, USA): Bruce W. Bode, MD, FACE. Barbara Davis Center for Diabetes (Aurora, Colorado, USA): Satish K. Garg, MD, Christie Beatson, CDCES. Barbara Davis Center for Childhood Diabetes (Aurora, Colorado, USA): Gregory P. Forlenza, MD. Children’s Minnesota (St. Paul, Minnesota, USA): M. Jennifer Abuzzahab, MD. Endocrine Research Solutions (Roswell, Georgia, USA): John H. Reed, MD. International Diabetes Center (Minneapolis, Minnesota, USA): Anders L. Carlson, MD. Joslin Diabetes Center at Harvard Medical School (Boston, Massachusetts, USA): Lori Laffel, MD, MPH, Kerri Milaszewski, RN, CDCES, Laurie Higgins, RD, CDCES. Medical Investigations, Inc. (Little Rock, Arkansas, USA): James R. Thrasher, MD, FACE. MultiCare Institute for Research & Innovation (Tacoma, Washington, USA): Bhuvana Sunil, MD, Rebekah Schaefer. Northwell Health (New Hyde Park, New York, USA): Benjamin Nwosu, MD, FAAP, Margaret Pellizzari, RN, Rashida Talib, MD, Nessa Wardak, BS. NYU Langone Hospital (Long Island, New York, USA): Siham Accacha, MD. Rainier Clinical Research Center (Renton, Washington, USA): Frances E. Broyles, MD. Sansum Diabetes Research Institute (Santa Barbara, California, USA): Kristin Castorino, DO, BC-ADM. Seattle Children’s Hospital (Seattle, Washington, USA): Catherine Pihoker, MD. Stanford University (Stanford, California, USA): Rayhan A. Lal, MD, Bailey Suh, Marci Morgan, Eliana Frank, Ryan Kingman, Lydia Liu, Kailee Kingston. Texas Diabetes and Endocrinology (Round Rock, Texas, USA): Luis Casaubon, MD. The Children’s Hospital of Philadelphia (Philadelphia, Pennsylvania, USA): Brynn Marks, MD, MSHPEd, Seema Meighan, DNP, CPNP-PC, MPH. University of California-San Francisco (San Francisco, California, USA): Laya Ekhlaspour, MD, Sonali Belapurkar, MD, Eda Cengiz, MD, Jasmine Hosseinipour, Avani Narayan. University of South Florida (Tampa, Florida, USA): Dorothy I. Shulman, MD, Janet Rodriguez, RN, CDE, Elizabeth Doble, MA. University Hospitals Cleveland Medical Center (Cleveland, Ohio, USA): Sarah A. MacLeish, DO, Terri Casey, RN, CDCES, Wendy Campbell, RN, CDCES, Jamie Wood, MD, Ramon Adams. Yale School of Medicine (New Haven, Connecticut, USA): Jennifer L. Sherr, MD, PhD, Laura M. Nally, MD, Kate Weyman, APRN, FNP-C, CDCES, Eileen Tichy, PA, Amy Steffen, BSN, Melinda Zgorski, BSN.

## Supplemental Material

sj-docx-1-dtt-10.1177_15209156251368928 — Supplemental material for Safety and Glycemic Outcomes of the MiniMed 780G System with a Disposable All-in-One Sensor Supplemental material, sj-docx-1-dtt-10.1177_15209156251368928 for Safety and Glycemic Outcomes of the MiniMed 780G System with a Disposable All-in-One Sensor by
