# Supplementary material for: Safety and Glycemic Outcomes of the MiniMed 780G System with a Disposable All-in-One Sensor
Source: Diabetes Technol Ther. Author manuscript; Available in PMC 2026 Feb 17. (PMC12911630; doi:10.1177/15209156251368928)
Supplement: Supplement [file NIHMS2139672-supplement-Supplement.docx]

# **Supplemental Materials**

## **Supplemental S1.** Study Flow


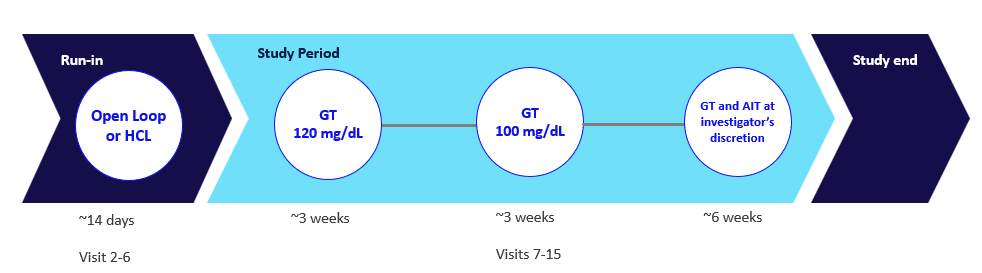


## **Supplemental S2.** Inclusion and Exclusion Criteria

| **Inclusion Criteria** | **Exclusion Criteria** |
| --- | --- |
| 1. Age 7 - 80 years at time of screening. 2. Has a clinical diagnosis of type 1 diabetes:    1. 14 – 80 years of age: A clinical diagnosis of type 1 diabetes for 2 years or more as determined via medical record or source documentation by an individual qualified to make a medical diagnosis.    2. 7 – 13 years of age: A clinical diagnosis of type 1 diabetes for 1 year or more as determined via medical record or source documentation by an individual qualified to make a medical diagnosis. 3. Does not require a legally authorized representative to consent on their behalf due to mental or intellectual disability. 4. Subject or parent/caregiver is literate and able to read the language offered in the pump or pump materials. 5. Subject and/or legally authorized representative is willing to provide informed consent for participation. 6. Is willing to perform fingerstick blood glucose measurements as needed. 7. Is willing to wear the system continuously throughout the study. 8. Must have a minimum daily insulin requirement (Total Daily Dose) of greater than or equal to 8 units. 9. Has a Glycosylated hemoglobin (A1C) less than 10% (as processed by Central Lab) at time of screening visit.   **Note:** All A1C blood specimens will be sent to and tested by a National Glycohemoglobin Standardization Program (NGSP) certified Central Laboratory. A1C testing must follow NGSP standards.   1. Has thyroid-stimulating hormone (TSH) in the normal range OR if the TSH is out of normal reference range the Free T3 is below or within the lab’s reference range and Free T4 is within the normal reference range. 2. Uses pump therapy for greater than 6 months prior to screening (with or without CGM experience). 3. Is willing to upload data from the study pump, must have Internet access, and a computer system, or compatible smartphone that meets the requirements for uploading the study pump. 4. Is willing to take one of the following insulins and can financially support the use of insulin preparations as required by the study:    1. Humalog (insulin lispro injection)    2. NovoLog (insulin aspart injection)    3. Admelog (insulin lispro injection) | 1. Has a history of 2 or more episodes of severe hypoglycemia, which resulted in any the following during the 6 months prior to screening:    1. Medical assistance (i.e., Paramedics, Emergency Room [ER] or Hospitalization)    2. Coma    3. Seizures 2. Has been hospitalized or has visited the ER in the 6 months prior to screening resulting in a primary diagnosis of uncontrolled diabetes. 3. Has had DKA in the last 6 months prior to screening visit. 4. Will not tolerate tape adhesive in the area of sensor placement as assessed by a qualified individual. 5. Has any unresolved adverse skin condition in the area of sensor placement (e.g., psoriasis, dermatitis herpetiformis, rash, Staphylococcus infection). 6. Is female of child-bearing potential and result of pregnancy test is positive at screening. 7. Is sexually active female of child-bearing potential and is not using a form of contraception deemed reliable by the investigator. 8. Is female and plans to become pregnant during the course of the study. 9. Is being treated for hyperthyroidism at time of screening. 10. Has diagnosis of adrenal insufficiency. 11. Has taken any oral, injectable, or intravenous (IV) glucocorticoids within 8 weeks from time of screening visit, or plans to take any oral, injectable, or IV glucocorticoids during the course of the study. 12. Is using hydroxyurea at time of screening or plans to use it during the study. 13. Is actively participating in an investigational study (drug or device) wherein he/she has received treatment from an investigational study drug or investigational study device in the last 2 weeks. 14. Has used a MiniMed™ 780G pump prior to screening. 15. Is currently abusing illicit drugs. 16. Is currently abusing marijuana. 17. Is currently abusing prescription drugs. 18. Is currently abusing alcohol. 19. Using pramlintide (Symlin), DPP-4 inhibitor, liraglutide (Victoza or other GLP-1 agonists), metformin, canagliflozin (Invokana or other SGLT2 inhibitors) at time of screening. 20. Has a history of visual impairment which would not allow subject to participate in the study and perform all study procedures safely, as determined by the investigator. 21. Has elective surgery planned that requires general anesthesia during the course of the study. 22. Has sickle cell disease, hemoglobinopathy; or has received red blood cell transfusion or erythropoietin within 3 months prior to time of screening. 23. Plans to receive red blood cell transfusion or erythropoietin over the course of study participation. 24. Is diagnosed with current eating disorder such as anorexia or bulimia. 25. Has been diagnosed with chronic kidney disease that results in chronic anemia. 26. Has a hematocrit that is below the normal reference range of lab used. 27. Is on dialysis. 28. Has serum creatinine of >2 mg/dL. 29. Has celiac disease that is not adequately treated as determined by the investigator. 30. Has had any of the following cardiovascular events within 1 year of screening: myocardial infarction, unstable angina, coronary artery bypass surgery, coronary artery stenting, transient ischemic attack, cerebrovascular accident, angina, congestive heart failure, or ventricular rhythm disturbances. 31. Has had history of cardiovascular event 1 year or more from the time of screening without     1. a normal EKG and stress test within 6 months prior to screening or during screening or     2. clearance from a qualified physician prior to receiving the study devices if there is an abnormal EKG or stress test. 32. Has 3 or more cardiovascular risk factors listed below without a normal EKG within 6 months prior to screening or during screening or clearance from a qualified physician if there is an abnormal EKG:     - Age >35 years     - Type 1 diabetes of >15 years’ duration     - Presence of any additional risk factor for coronary artery disease     - Presence of microvascular disease (proliferative retinopathy or nephropathy, including microalbuminuria)     - Presence of peripheral vascular disease     - Presence of autonomic neuropathy 33. Is a member of the research staff involved with the study. 34. Is a Medtronic Diabetes employee or their immediate family member (excluding adult children and/or adult siblings). |

## **Supplemental S3.** Study participant disposition


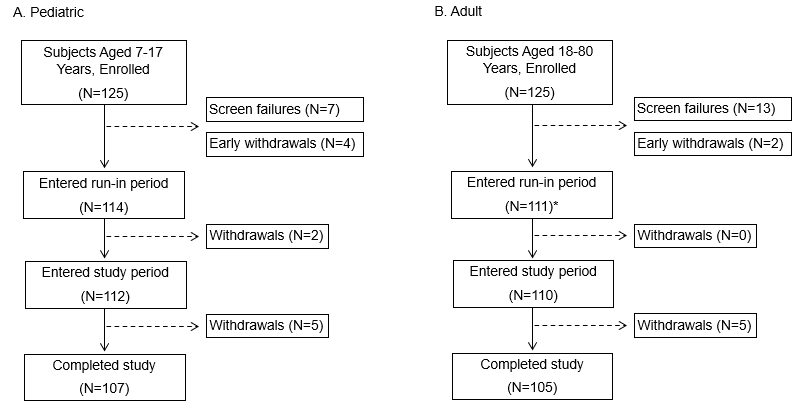


**Supplemental S3 Caption.** The number of enrolled (A.) pediatric participants was N=125 and the intention-to-treat population included N=112 participants. The number of enrolled (B.) adult participants was N=125 and the intention-to-treat population included N=110 participants. *One subject was a screen failure but entered the run-in period.
